# Supplementary material for: Preliminary Assessment of Bespoke (‘X-tec’) Silica Particles for IRS Applications
Source: Insects. 2025 Sep 5;16(9):937. doi: 10.3390/insects16090937 (PMC12470417; doi:10.3390/insects16090937)
Supplement: Supplementary file 1 [file insects-16-00937-s001.zip › Tables and graphs.pdf]

Table S1. 1-week post spray: 60-minute knockdown (KD) and 24–120-hour mortality of *An. gambiae* s.s. Kisumu exposed in cone bioassays to glazed tiles sprayed with 10% X-tec silica at three application rates (30, 60 and 90 a.i. mg/m2), unformulated silica, and an untreated control tile. Replicates were pooled therefore no standard deviations could be calculated.

Date	Time point	Colony	Substrate	Treatment	N	Reps	Average % knockdown/mortality					
							60 min	24 hr	48 hr	72 hr	96 hr	120 hr
03/09/21	1 week	Kisumu	Glazed tiles	Untreated	39	1	0.00	5.13	12.82	15.38	23.08	23.08
03/09/21	1 week	Kisumu	Glazed tiles	NIRS-SB-0 Xtec silica	39	1	2.56	2.56	12.82	17.95	23.08	23.08
03/09/21	1 week	Kisumu	Glazed tiles	10% Xtec Silia Clothianidin 30mg/m^2	40	1	2.50	87.50	97.50	100.00	100.00	100.00
03/09/21	1 week	Kisumu	Glazed tiles	10% Xtec Silia Clothianidin 60mg/m^2	40	1	20.00	100.00	100.00	100.00	100.00	100.00
03/09/21	1 week	Kisumu	Glazed tiles	10% Xtec Silia Clothianidin 90mg/m^2	35	1	17.14	100.00	100.00	100.00	100.00	100.00

Abbreviations: N = number of mosquitoes, Rep = Number of tile replicates, min = minutes, hr = hours.

Table S2. 1-week post spray: 60-minute knockdown (KD) and 24–120-hour mortality of *An. gambiae* s.s. Kisumu exposed in a cone bioassays to unglazed tiles sprayed with 10% X-tec silica at three application rates (30, 60 and 90 a.i. mg/m2), unformulated silica, and an untreated control tile.

Date	Time point	Colony	Substrate	Treatment	N	Reps	Average % knockdown/mortality (SD)					
							60 min	24 hr	48 hr	72 hr	96 hr	120 hr
03/09/21	1 week	Kisumu	Unglazed tiles	Untreated	36	4	0.00 (0.00)	2.50 (5.00)	2.50 (5.00)	10.83 (9.09)	13.61 (10.75)	13.61 (10.75)
03/09/21	1 week	Kisumu	Unglazed tiles	NIRS-SB-0 Xtec silica	41	4	2.50 (5.00)	2.50 (5.00)	9.77 (8.18)	9.77 (8.18)	12.27 (12.65)	12.27 (12.65)
03/09/21	1 week	Kisumu	Unglazed tiles	10% Xtec Silia Clothianidin 30mg/m2	40	4	0.00 (0.00)	12.37 (11.23)	42.93 (28.14)	55.30 (27.20)	58.08 (26.27)	58.08 (26.27)
03/09/21	1 week	Kisumu	Unglazed tiles	10% Xtec Silia Clothianidin 60mg/m2	40	4	2.50 (5.00)	73.54 (25.44)	100.00 (0.00)	100.00 (0.00)	100.0 (0.00)	100.00 (0.00)
03/09/21	1 week	Kisumu	Unglazed tiles	10% Xtec Silia Clothianidin 90mg/m2	41	4	6.82 (13.64)	97.50 (5.00)	100.00 (0.00)	100.00 (0.00)	100.00 (0.00)	100.00 (0.00)

Abbreviations: N = number of mosquitoes, Rep = Number of tile replicates, min = minutes, hr = hours, SD = Standard deviation.

Table S3. 8-months post spray: 60-minute knockdown (KD) and 24–120-hour mortality of *An. gambiae* s.s. Kisumu exposed in a cone bioassays to glazed tiles sprayed with 10% X-tec silica at three application rates (30, 60 and 90 a.i. mg/m2), unformulated silica, and an untreated control tile.

Date	Time point	Colony	Substrate	Treatment	N	Reps	Average % knockdown/mortality (SD)					
							60 min	24 hr	48 hr	72 hr	96 hr	120 hr
11/05/22	8 month	Kisumu	Glazed tiles	Untreated	40	4	0.00 (0.00)	0.00 (0.00)	5.28 (6.11)	5.28 (6.11)	9.82 (7.48)	9.82 (7.48)
11/05/22	8 month	Kisumu	Glazed tiles	NIRS-SB-0 Xtec silica	19	2	5.56 (7.86)	16.67 (23.57)	21.67 (16.50)	21.67 (16.50)	21.67 (16.50)	21.67 (16.50)
11/05/22	8 month	Kisumu	Glazed tiles	10% Xtec Silia Clothianidin 30mg/m2	20	2	35.00 (7.07)	70.00 (42.43)	100.00 (0.00)	100.00 (0.00)	100.00 (0.00)	100.00 (0.00)
11/05/22	8 month	Kisumu	Glazed tiles	10% Xtec Silia Clothianidin 60mg/m2	20	2	25.00 (7.07)	100.00 (0.00)	100.00 (0.00)	100.00 (0.00)	100.00 (0.00)	100.00 (0.00)
11/05/22	8 month	Kisumu	Glazed tiles	10% Xtec Silia Clothianidin 90mg/m2	21	2	5.00 (7.07)	100.00 (0.00)	100.00 (0.00)	100.00 (0.00)	100.00 (0.00)	100.00 (0.00)

Abbreviations: N = number of mosquitoes, Rep = Number of tile replicates, min = minutes, hr = hours, SD = Standard deviation.

Table S4. 8-months post spray: 60-minute knockdown (KD) and 24–120-hour mortality (96-hour mortality was not recorded) of *An. gambiae* s.s. Kisumu exposed in a cone bioassays to unglazed tiles sprayed with 10% X-tec silica at three application rates (30, 60 and 90 a.i. mg/m2), unformulated silica, and an untreated control tile.

Date	Time point	Colony	Substrate	Treatment	N	Reps	Average % knockdown/mortality (SD)					
							60 min	24 hr	48 hr	72 hr	96 hr	120 hr
24/05/22	8 month	Kisumu	Unglazed tiles	Untreated	40	4	0.00 (0.00)	5.00 (5.77)	7.50 (5.00)	7.50 (5.00)	N/A	15.00 (5.77)
24/05/22	8 month	Kisumu	Unglazed tiles	NIRS-SB-0 Xtec silica	20	2	0.00 (0.00)	0.00 (0.00)	0.00 (0.00)	0.00 (0.00)	N/A	0.00 (0.00)
24/05/22	8 month	Kisumu	Unglazed tiles	10% Xtec Silia Clothianidin 30mg/m2	20	2	10.00 (14.14)	10.00 (14.14)	10.00 (14.14)	10.00 (14.14)	N/A	25.00 (21.21)
24/05/22	8 month	Kisumu	Unglazed tiles	10% Xtec Silia Clothianidin 60mg/m2	19	2	0.00 (0.00)	31.67 (2.36)	57.78 (3.14)	89.44 (0.79)	N/A	100.00 (0.00)
24/05/22	8 month	Kisumu	Unglazed tiles	10% Xtec Silia Clothianidin 90mg/m2	18	2	6.25 (8.84)	51.25 (15.91)	95.00 (7.07)	100.00 (0.00)	N/A	100.00 (0.00)

Abbreviations: N = number of mosquitoes, Rep = Number of tile replicates, min = minutes, hr = hours, SD = Standard deviation.
